# Mixed finite elements for global tide models

**DOI:** 10.1007/s00211-015-0748-z

**Published:** 2015-07-10

**Authors:** Colin J. Cotter, Robert C. Kirby

**Affiliations:** 1 Imperial College London, South Kensington Campus, London, SW7 2AZ UK; 2 Department of Mathematics, Baylor University, One Bear Place #97328, Waco, TX 76798-7328 USA

**Keywords:** 65M12, 65M60, 35Q86

## Abstract

We study mixed finite element methods for the linearized rotating shallow water equations with linear drag and forcing terms. By means of a strong energy estimate for an equivalent second-order formulation for the linearized momentum, we prove long-time stability of the system without energy accumulation—the geotryptic state. A priori error estimates for the linearized momentum and free surface elevation are given in $L^2$ as well as for the time derivative and divergence of the linearized momentum. Numerical results confirm the theoretical results regarding both energy damping and convergence rates.

## Introduction

Finite element methods are attractive for modelling the world’s oceans since implemention with triangular cells provides a means to accurately represent coastlines and topography [36]. In the last decade or so, there has been much discussion about the best choice of mixed finite element pairs to use as the horizontal discretization for atmosphere and ocean models. In particular, much attention has been paid to the properties of numerical dispersion relations obtained when discretizing the rotating shallow water equations [5, 6, 10, 22, 30–33]. In this paper we take a different angle, and study the behavior of discretizations of forced-dissipative rotating shallow-water equations, which are used for predicting global barotropic tides. The main point of interest here is whether the discrete solutions approach the correct long-time solution in response to quasi-periodic forcing. In particular, we study the behavior of the linearized energy. Since this energy only controls the divergent part of the solution, as we shall see later, it is important to choose finite element spaces where there is a natural discrete Helmholtz decomposition, and where the Coriolis term projects the divergent and divergence-free components of vector fields correctly onto each other. Hence, we choose to concentrate on the mimetic, or compatible, finite element spaces (i.e. those which arise naturally from the finite element exterior calculus [1]) which were proposed for numerical weather prediction in [7]. In that paper, it was shown that the discrete equations have an exactly steady geostrophic state (a solution in which the Coriolis term balances the pressure gradient) corresponding to each of the divergence-free velocity fields in the finite element space; this approach was extended to develop finite element methods for the nonlinear rotating shallow-water equations on the sphere that can conserve energy, enstrophy and potential vorticity [8, 26, 29]. Here, we shall make use of the discrete Helmholtz decomposition in order to show that mixed finite element discretizations of the forced-dissipative linear rotating shallow-water equations have the correct long-time energy behavior. Since we are studying linear equations, these energy estimates then provide finite time error bounds.

Predicting past and present ocean tides is important because they have a strong impact on sediment transport and coastal flooding, and hence are of interest to geologists. Recently, tides have also received a lot of attention from global oceanographers since breaking internal tides provide a mechanism for vertical mixing of temperature and salinity that might sustain the global ocean circulation [12, 27]. A useful tool for predicting tides are the rotating shallow water equations, which provide a model of the barotropic (i.e., depth-averaged) dynamics of the ocean. When modelling global barotropic tides away from coastlines, the nonlinear advection terms are very weak compared to the drag force, and a standard approach is to solve the linear rotating shallow-water equations with a parameterized drag term to model the effects of bottom friction, as described in [21]. This approach can be used on a global scale to set the boundary conditions for a more complex regional scale model, as was done in [14], for example. Various additional dissipative terms have been proposed to account for other dissipative mechanisms in the barotropic tide, due to baroclinic tides, for example[16].

As mentioned above, finite element methods provide useful discretizations for tidal models since they can be used on unstructured grids which can seamless couple global tide structure with local coastal dynamics. A discontinuous Galerkin approach was developed in [34], whilst continuous finite element approaches have been used in many studies ([11, 18, 23], for example). The lowest order Raviart–Thomas element for velocity combined with $P_0$ for height was proposed for coastal tidal modeling in [35]; this pair fits into the framework that we discuss in this paper.

In this paper we will restrict attention to the linear bottom drag model as originally proposed in [21]. We are aware that the quadratic law is more realistic, but the linear law is more amenable to analysis and we believe that the correct energy behavior of numerical methods in this linear setting already rules out many methods which are unable to correctly represent the long-time solution which is in geotryptic balance (the extension to geostrophic balance of the three way balance between Coriolis, the pressure gradient and the dissipative term). In the presence of quasiperiodic time-varying tidal forcing, the equations have a time-varying attracting solution that all solutions converge to as $t\rightarrow \infty$. In view of this, we prove the following results which are useful to tidal modellers (at least, for the linear law):

1. For the mixed finite element methods that we consider, the spatial semidiscretization also has an attracting solution in the presence of time-varying forcing.
2. This attracting solution converges to time-varying attracting solution of the unapproximated equations.

Global problems require tidal simulation on manifolds rather than planar domains. For simplicity, our description and analysis will follow the latter case. However, our numerical results include the former case. Recently, Holst and Stern [15] have demonstrated that finite element analysis on discretized manifolds can be handled as a variational crime. We summarize these findings and include an appendix at the end demonstrating how to apply their techniques to our own case. This suggests that the extension to manifolds presents technicalities rather than difficulties to the analysis we provide here.

Although our present work focuses squarely on the shallow water equations, we believe that many of our results will apply to other hyperbolic systems with damping. For example, the model we consider is just the damped acoustic wave equation plus the Coriolis term. Our techniques should extend to other settings where function spaces have discrete Helmholtz decompositions, most notably damped electromagnetics or elastodynamics.

Also, we point out that our main aim here is the theoretical analysis of the damped system. This is the first such analysis of which we are aware demonstrating the strong damping and hence optimal long-time error bounds. We do not assert whether similar results hold for other discretizations, just that they are unkown. While an extended experimental and/or theoretical study of these properties for a wide range of discretizations could be a fruitful project for the tide modelling community, it is beyond the scope of the present work. We do point out that the lowest-order Raviart–Thomas element has been previously proposed for tidal modeling [35], and our framework covers this case as well as the extension to higher-order methods and other mixed spaces.

The rest of this paper is organised as follows. In Sect. 2 we describe the finite element modelling framework which we will analyse. In Sect. 3 we provide some mathematical preliminaries. In Sect. 4 we derive energy stability estimates for the finite element tidal equations. In Sect. 5 we use these energy estimates to obtain error bounds for our numerical solution. Appendix A includes the discussion of embedded manifolds.

## Description of finite element tidal model

We start with the nondimensional linearized rotating shallow water model with linear drag and forcing on a (possibly curved) two dimensional surface ${\varOmega }$, given by

1 $$\begin{aligned}&u_t + \frac{f}{\epsilon } u^\perp + \frac{\beta }{\epsilon ^2} \nabla \left( \eta - \eta ^\prime \right) + C u = 0, \nonumber \\&\eta _t + \nabla \cdot \left( H u \right) = 0, \end{aligned}$$

where *u* is the nondimensional two dimensional velocity field tangent to ${\varOmega }$, $u^\perp =(-u_2,u_1)$ is the velocity rotated by $\pi /2$, $\eta$ is the nondimensional free surface elevation above the height at state of rest, $\nabla \eta '$ is the (spatially varying) tidal forcing, $\epsilon$ is the Rossby number (which is small for global tides), *f* is the spatially-dependent non-dimensional Coriolis parameter which is equal to the sine of the latitude (or which can be approximated by a linear or constant profile for local area models), $\beta$ is the Burger number (which is also small), *C* is the (spatially varying) nondimensional drag coefficient and *H* is the (spatially varying) nondimensional fluid depth at rest, and $\nabla$ and $\nabla \cdot$ are the intrinsic gradient and divergence operators on the surface ${\varOmega }$, respectively.

We will work with a slightly generalized version of the forcing term, which will be necessary for our later error analysis. Instead of assuming forcing of the form $\frac{\beta }{\epsilon ^2} \nabla \eta ^\prime$, we assume some $F \in L^2$, giving our model as

2 $$\begin{aligned}&u_t + \frac{f}{\epsilon } u^\perp + \frac{\beta }{\epsilon ^2} \nabla \eta + C u = F, \nonumber \\&\eta _t + \nabla \cdot \left( H u \right) = 0. \end{aligned}$$

It also becomes useful to work in terms of the linearized momentum $\widetilde{u} = H u$ rather than velocity. After making this substitution and dropping the tildes, we obtain

3 $$\begin{aligned} \begin{aligned}&\displaystyle \frac{1}{H}u_t + \frac{f}{H\epsilon } u^\perp + \frac{\beta }{\epsilon ^2} \nabla \eta + \frac{C}{H} u = F,\\&\displaystyle \eta _t + \nabla \cdot u = 0. \end{aligned} \end{aligned}$$

A natural weak formulation of these equations is to seek $u \in H(\mathrm {div})$ and $\eta \in L^2$ so that

4 $$\begin{aligned}&\displaystyle \left( \frac{1}{H}u_t , v \right) \!+\! \frac{1}{\epsilon } \left( \frac{f}{H} u^\perp , v \right) - \frac{\beta }{\epsilon ^2} \left( \eta , \nabla \cdot v \right) \!+\! \left( \frac{C}{H} u , v \right) \!=\! \left( F, v \right) \!, \quad \forall v \in H(\mathrm {div}), \nonumber \\&\displaystyle \quad \left( \eta _t , w \right) + \left( \nabla \cdot u , w \right) = 0, \quad \forall w \in L^2. \end{aligned}$$

We now develop mixed discretizations with $V_h \subset H(\mathrm {div})$ and $W_h \subset L^2$. Conditions on the spaces are the commuting projection and divergence mapping $V_h$ onto $W_h$. We define $u_h \subset V_h$ and $\eta _h \subset W_h$ as solutions of the discrete variational problem

5 $$\begin{aligned}&\displaystyle \left( \frac{1}{H}u_{h,t} , v_h \right) + \frac{1}{\epsilon } \left( \frac{f}{H} u_h^\perp , v_h \right) - \frac{\beta }{\epsilon ^2} \left( \eta _h , \nabla \cdot v_h \right) + \left( \frac{C}{H} u_h , v_h \right) = \left( F , v_h \right) , \nonumber \\&\displaystyle \quad \left( \eta _{h,t} , w_h \right) + \left( \nabla \cdot u_h , w_h \right) = 0. \end{aligned}$$

We will eventually obtain stronger estimates by working with an equivalent second-order form. If we take the time derivative of the first equation in (5) and use the fact that $\nabla \cdot V_h = W_h$, we have

6 $$\begin{aligned} \left( \frac{1}{H}u_{h,tt} , v_h \right) \!+\! \frac{1}{\epsilon } \left( \frac{f}{H} u_{h,t}^\perp , v_h \right) \!+\! \frac{\beta }{\epsilon ^2} \left( \nabla \cdot u_h , \nabla \cdot v_h \right) \!+\! \left( \frac{C}{H} u_{h,t} , v_h \right) = \left( \widetilde{F} , v_h \right) ,\nonumber \\ \end{aligned}$$

where $\widetilde{F} = F_t$. This is a restriction of

7 $$\begin{aligned} \left( \frac{1}{H}u_{tt} , v \right) + \frac{1}{\epsilon } \left( \frac{f}{H} u_{t}^\perp , v \right) + \frac{\beta }{\epsilon ^2} \left( \nabla \cdot u , \nabla \cdot v \right) + \left( \frac{C}{H} u_t , v \right) = \left( \widetilde{F} , v_h \right) , \end{aligned}$$

which is the variational form of

8 $$\begin{aligned} \frac{1}{H} u_{tt} + \frac{f}{H} u^\perp _t - \frac{\beta }{\epsilon ^2} \nabla \left( \nabla \cdot u \right) + \frac{C}{H} u_t = \widetilde{F}, \end{aligned}$$

to the mixed finite element spaces. Note that here, we have taken the time derivative at the discrete level, and so this is an equivalent formulation to (5), making use of the compatible spaces, and so the problems associated with discretizing the equations in wave equation form (such as the methods described in [19, 25]) do not arise.

We have already discussed mixed finite elements’ application to tidal models in the geophysical literature, but this work also builds on existing literature for mixed discretization of the acoustic equations. The first such investigation is due to Geveci [13], where exact energy conservation and optimal error estimates are given for the semidiscrete first-order form of the model wave equation. Later analysis [9, 17] considers a second order in time wave equation with an auxillary flux at each time step. In [20], Kirby and Kieu return to the first-order formulation, giving additional estimates beyond [13] and also analyzing the symplectic Euler method for time discretization. From the standpoint of this literature, our model (3) appends additional terms for the Coriolis force and damping to the simple acoustic model. We restrict ourselves to semidiscrete analysis in this work, but pay careful attention the extra terms in our estimates, showing how study of an equivalent second-order equation in $H(\mathrm {div})$ proves proper long-term behavior of the model.

## Mathematical preliminaries

For the velocity space $V_h$, we will work with standard $H(\mathrm {div})$ mixed finite element spaces on triangular elements, such as Raviart–Thomas (RT), Brezzi–Douglas–Marini (BDM), and Brezzi–Douglas–Fortin–Marini (BDFM) [3, 4, 28]. We label the lowest-order Raviart–Thomas space with index $k=1$, following the ordering used in the finite element exterior calculus [1]. Similarly, the lowest-order Brezzi–Douglas–Fortin–Marini and Brezzi–Douglas–Marini spaces correspond to $k=1$ as well. We will always take $W_h$ to consist of piecewise polynomials of degree $k-1$, not constrained to be continuous between cells. In the case of domains with boundaries, we require the strong boundary condition $u\cdot n = 0$ on all boundaries.

In the main part of this paper we shall present results assuming that the domain is a subset of $\mathbb {R}^2$, i.e. flat geometry. In the Appendix, we describe how to extend these results to the case of embedded surfaces in $\mathbb {R}^3$.

Throughout, we shall let $\left\| \cdot \right\|$ denote the standard $L^2$ norm. We will frequently work with weighted $L^2$ norms as well. For a positive-valued weight function $\kappa$, we define the weighted $L^2$ norm

9 $$\begin{aligned} \left\| g \right\| _{\kappa }^2 = \int _{\varOmega } \kappa \left| g \right| ^2dx. \end{aligned}$$

If there exist positive constants $\kappa _*$ and $\kappa ^*$ such that $0 < \kappa _* \le \kappa \le \kappa ^* < \infty$ almost everywhere, then the weighted norm is equivalent to the standard $L^2$ norm by

10 $$\begin{aligned} \sqrt{\kappa _*} \left\| g \right\| \le \left\| g \right\| _{\kappa } \le \sqrt{\kappa ^*} \left\| g \right\| . \end{aligned}$$

A Cauchy–Schwarz inequality

11 $$\begin{aligned} (\kappa g_1 , g_2) \le \left\| g_1 \right\| _{\kappa } \left\| g_2 \right\| _{\kappa } \end{aligned}$$

holds for the weighted inner product, and we can also incorporate weights into Cauchy–Schwarz for the standard $L^2$ inner product by

12 $$\begin{aligned} (g_1,g_2) = \left( \sqrt{\kappa } g_1 , \frac{1}{\sqrt{\kappa }} g_2\right) \le \left\| g_1 \right\| _{\kappa } \left\| g_2 \right\| _{\frac{1}{\kappa }}. \end{aligned}$$

We refer the reader to references such as [3] for full details about the particular definitions and properties of these spaces, but here recall several facts essential for our analysis. For all velocity spaces $V_h$ we consider, the divergence maps $V_h$ onto $W_h$. Also, the spaces of interest all have a projection, $\Pi : H(\mathrm {div})\rightarrow V_h$ that commutes with the $L^2$ projection $\pi$ into $W_h$:

13 $$\begin{aligned} \left( \nabla \cdot \Pi u , w_h \right) = \left( \pi \nabla \cdot u , w_h \right) \end{aligned}$$

for all $w_h \in W_h$ and any $u \in H(\mathrm {div})$. We have the error estimate

14 $$\begin{aligned} \left\| u - \Pi u \right\| \le C_{\Pi } h^{k+\sigma } \left| u \right| _{k} \end{aligned}$$

when $u \in H^{k+1}$. Here, $\sigma = 1$ for the BDM spaces but $\sigma =0$ for the RT or BDFM spaces. The projection also has an error estimate for the divergence

15 $$\begin{aligned} \left\| \nabla \cdot \left( u - \Pi u \right) \right\| \le C_\Pi h^{k} \left| \nabla \cdot u \right| _{k} \end{aligned}$$

for all the spaces of interest, whilst the pressure projection has the error estimate

16 $$\begin{aligned} \left\| \eta - \pi \eta \right\| \le C_{\pi } h^k \left| \eta \right| _{k}. \end{aligned}$$

Here, $C_\Pi$ and $C_\pi$ are positive constants independent of *u*, $\eta$, and *h*, although not necessarily of the shapes of the elements in the mesh.

We will utilize a Helmholtz decomposition of $H(\mathrm {div})$ under a weighted inner product. For a very general treatment of such decompositions, we refer the reader to [2]. For each $u \in V$, there exist unique vectors $u^D$ and $u^S$ such that $u = u^D + u^S$, $\nabla \cdot u^S = 0$, and also $\left( \frac{1}{H} u^D , u^S \right) = 0$. That is, $H(\mathrm {div})$ is decomposed into the direct sum of solenoidal vectors, which we denote by

17 $$\begin{aligned} \mathcal {N} \left( \nabla \cdot \right) = \left\{ u \in V : \nabla \cdot u = 0 \right\} , \end{aligned}$$

and its orthogonal complement under the $\left( \frac{1}{H} \cdot , \cdot \right)$ inner product, which we denote by

18 $$\begin{aligned} \mathcal {N} \left( \nabla \cdot \right) ^\perp = \left\{ u \in V : \left( \frac{1}{H} u , v \right) = 0, \ \forall v \in \mathcal {N} \left( \nabla \cdot \right) \right\} . \end{aligned}$$

Functions in $\mathcal {N} \left( \nabla \cdot \right) ^\perp$ satisfy a generalized Poincaré-Friedrichs inequality, that there exists some $C_P$ such that

19 $$\begin{aligned} \left\| u^D \right\| _{\frac{1}{H}} \le C_P \left\| \nabla \cdot u^D \right\| _{\frac{1}{H}}. \end{aligned}$$

We may also use norm equivalence to write this as

20 $$\begin{aligned} \left\| u^D \right\| _{\frac{1}{H}} \le \frac{C_P}{\sqrt{H_*}} \left\| \nabla \cdot u^D \right\| . \end{aligned}$$

Because our mixed spaces $V_h$ are contained in $H(\mathrm {div})$, the same decompositions can be applied, and the Poincaré-Friedrichs inequality holds with a constant no larger than $C_p$.

## Energy estimates

In this section, we develop in stability estimates for our system, obtained by energy techniques. Supposing that there is no forcing or damping ($F = C = 0$), we pick $v_h = u_h$ and $w_h =\frac{\beta }{\epsilon ^2} \eta _h$ in (5), and find that

21 $$\begin{aligned} \begin{aligned}&\displaystyle \left( \frac{1}{H} u_{h,t} , u_h \right) + \frac{1}{\epsilon } \left( \frac{f}{H} u_h^\perp , u_h \right) - \frac{\beta }{\epsilon ^2}\left( \eta _h , \nabla \cdot u_h \right) = 0, \\&\displaystyle \frac{\beta }{\epsilon ^2} \left( \eta _{h,t} , \eta _h \right) + \frac{\beta }{\epsilon ^2} \left( \nabla \cdot u_h , \eta _h \right) = 0. \end{aligned} \end{aligned}$$

Since $u_h^\perp \cdot u_h = 0$ pointwise, we add these two equations together to find

22 $$\begin{aligned} \frac{1}{2} \frac{d}{dt} \left\| u_h \right\| _{\frac{1}{H}}^2 + \frac{\beta }{2\epsilon ^2} \frac{d}{dt} \left\| \eta _h \right\| ^2 = 0. \end{aligned}$$

Hence, we have the following.

### **Proposition 1**

In the absence of damping or forcing, the quantity

23 $$\begin{aligned} E_1(t) = \frac{1}{2} \left\| u_h \right\| _{\frac{1}{H}}^2 + \frac{\beta }{2\epsilon ^2} \left\| \eta _h \right\| ^2 \end{aligned}$$

is conserved exactly for all time.

Now suppose that $F = 0$ still but that $0 < C_* \le C \le C < \infty$ pointwise in ${\varOmega }$. The same considerations now lead to

24 $$\begin{aligned} \frac{1}{2} \frac{d}{dt} \left\| u_h \right\| _{\frac{1}{H}}^2 + \frac{\beta }{2\epsilon ^2} \frac{d}{dt} \left\| \eta _h \right\| ^2 + \left\| u_h \right\| _{\frac{C}{H}}^2 = 0, \end{aligned}$$

so that

### **Proposition 2**

In the absence of forcing, but with $0 < C_* \le C \le C < \infty$, the quantity $E_1(t)$ defined in (23) satisfies

$$\begin{aligned} \frac{d}{dt} E_1(t) \le 0. \end{aligned}$$

In the presence of forcing and dissipation, it is also possible to make estimates showing worst-case linear accumulation of the energy over time.

### **Proposition 3**

With nonzero *F*, we have that for all time *t*,

25 $$\begin{aligned} E_1(t) \le E_1(0) + \frac{1}{2C_*} \int _0^t \left\| F\left( \cdot , s \right) \right\| _{H}^2 ds \end{aligned}$$

### *Proof*

We choose $w_h$ and $v_h$ as without forcing, and find that

$$\begin{aligned} \frac{d}{dt} E_1(t) + \left\| u\left( \cdot , t \right) \right\| _{\frac{C}{H}}^2 = \left( F , u_h \right) . \end{aligned}$$

Cauchy–Schwarz, Young’s inequality, and norm equivalence give

$$\begin{aligned} \frac{d}{dt} E_1(t) + \frac{C_*}{2} \left\| u_h\left( \cdot , t \right) \right\| _{\frac{1}{H}}^2 \le \frac{1}{2C_*} \left\| F\left( \cdot , t \right) \right\| _{H}^2 \end{aligned}$$

The result follows by dropping the positive term from the left-hand side and integrating.

However, linear energy accumulation is not observed for actual tidal motion, so we expect a stronger result to hold. Turning to the second order equation (6), we begin with vanishing forcing and damping terms, putting $v_h = u_{h,t}$ to find

26 $$\begin{aligned} \left( \frac{1}{H}u_{h,tt} , u_{h,t} \right) + \frac{1}{\epsilon } \left( \frac{f}{H} u_{h,t}^\perp , u_{h,t} \right) + \frac{\beta }{\epsilon ^2} \left( \nabla \cdot u_h , \nabla \cdot u_{h,t} \right) = 0, \end{aligned}$$

which simplifies to

27 $$\begin{aligned} \frac{1}{2} \frac{d}{dt} \left\| u_{h,t} \right\| _{\frac{1}{H}}^2 + \frac{\beta }{2\epsilon ^2} \frac{d}{dt} \left\| \nabla \cdot u_h \right\| ^2 = 0, \end{aligned}$$

so that the quantity

28 $$\begin{aligned} E(t) = \frac{1}{2} \left\| u_{h,t} \right\| _{\frac{1}{H}}^2 + \frac{\beta }{2\epsilon ^2} \left\| \nabla \cdot u_h \right\| ^2 \end{aligned}$$

is conserved exactly for all time.

If *C* is nonzero, we have that

29 $$\begin{aligned} \frac{1}{2} \frac{d}{dt} \left\| u_{h,t} \right\| _{\frac{1}{H}}^2 + \frac{\beta }{2\epsilon ^2} \frac{d}{dt} \left\| \nabla \cdot u_h \right\| ^2 + \left\| u_{h,t} \right\| _{\frac{C}{H}}^2 = 0, \end{aligned}$$

which implies that *E*(*t*) is nonincreasing, although with no particular decay rate.

Now, we develop more refined technique based on the Helmholtz decomposition that gives a much stronger damping result. We can write $u_h = u_h^D + u_h^S$ in the $\frac{1}{H}$-weighted decomposition. We let $0 < \alpha$ be a scalar to be determined later and let the test function *v* in (6) be $v_h = u_{h,t} + \alpha u^D_h$. This gives

30 $$\begin{aligned}&\left( \frac{1}{H}u_{h,tt} , u_{h,t} + \alpha u^D_h \right) + \frac{1}{\epsilon } \left( \frac{f}{H} u_{h,t}^\perp , u_{h,t} + \alpha u^D_h \right) \nonumber \\&\quad +\,\, \frac{\beta }{\epsilon ^2} \left( \nabla \cdot u_h , \nabla \cdot \left( u_{h,t} + \alpha u^D_h \right) \right) + \left( \frac{C}{H} u_{h,t} , u_{h,t} + \alpha u^D_h \right) = 0, \end{aligned}$$

and we rewrite the left-hand side so that

31 $$\begin{aligned}&\frac{1}{2} \frac{d}{dt} \left\| u_{h,t} \right\| _{\frac{1}{H}}^2 + \alpha \left( \frac{1}{H} u_{h,tt} , u_h^D \right) + \frac{\alpha }{\epsilon } \left( \frac{f}{H} u_{h,t}^\perp , u_h^D \right) \nonumber \\&\quad + \frac{\beta }{2 \epsilon ^2} \frac{d}{dt} \left\| \nabla \cdot u_h^D \right\| ^2 + \frac{\alpha \beta }{\epsilon ^2} \left\| \nabla \cdot u_h^D \right\| ^2 + \left\| u_{h,t} \right\| _{\frac{C}{H}}^2 + \alpha \left( \frac{C}{H} u_{h,t} , u_{h}^D \right) = 0.\qquad \quad \end{aligned}$$

We use the fact that

$$\begin{aligned} \frac{d}{dt} \left( \frac{1}{H} u_{h,t} , u_h^D \right) = \left( \frac{1}{H} u_{h,tt} , u_h^D \right) + \left( \frac{1}{H} u_{h,t} , u_{h,t}^d \right) \end{aligned}$$

and also that $u_h^S$ is $\frac{1}{H}$-orthogonal to $u_h^D$ to rewrite the left-hand side as

32 $$\begin{aligned}&\frac{d}{dt} \left[ \frac{1}{2} \left\| u_{h,t} \right\| _{\frac{1}{H}}^2 + \alpha \left( \frac{1}{H} u_{h,t} , u_h^D \right) + \frac{\beta }{2 \epsilon ^2} \left\| \nabla \cdot u_h^D \right\| ^2 \right] \nonumber \\&\quad + \frac{\alpha }{\epsilon } \left( \frac{f}{H} u_{h,t}^\perp , u_h^D \right) + \frac{\alpha \beta }{\epsilon ^2} \left\| \nabla \cdot u_h^D \right\| ^2 \nonumber \\&\quad + \left\| u_{h,t} \right\| _{\frac{C}{H}}^2 - \alpha \left\| u_{h,t}^D \right\| _{\frac{1}{H}}^2 + \alpha \left( \frac{C}{H} u_{h,t} , u_h^D \right) = 0. \end{aligned}$$

This has the form of an ordinary differential equation

33 $$\begin{aligned} A^\prime (t) + B(t) = 0, \end{aligned}$$

where

34 $$\begin{aligned} A(t) = \frac{1}{2} \left\| u_{h,t} \right\| _{\frac{1}{H}}^2 + \alpha \left( \frac{1}{H} u_{h,t} , u_h^D \right) + \frac{\beta }{2 \epsilon ^2} \left\| \nabla \cdot u_h^D \right\| ^2 \end{aligned}$$

and

35 $$\begin{aligned} B(t)= & {} \frac{\alpha }{\epsilon } \left( \frac{f}{H} u_{h,t}^\perp , u_h^D \right) + \frac{\alpha \beta }{\epsilon ^2} \left\| \nabla \cdot u_h^D \right\| ^2 \nonumber \\&+ \left\| u_{h,t} \right\| _{\frac{C}{H}}^2 - \alpha \left\| u_{h,t}^D \right\| _{\frac{1}{H}}^2 + \alpha \left( \frac{C}{H} u_{h,t} , u_h^D \right) . \end{aligned}$$

By showing that for suitably chosen $\alpha$, both *A*(*t*) and *B*(*t*) are comparable to *E*(*t*) defined in (28), we can obtain exponential damping of the energy.

### **Lemma 1**

Suppose that

36 $$\begin{aligned} \alpha \le \alpha _1 \equiv \frac{\sqrt{\beta H_*}}{2 C_p \epsilon }. \end{aligned}$$

Then

37 $$\begin{aligned} \frac{1}{2} E(t) \le A(t) \le \frac{3}{2} E(t). \end{aligned}$$

### *Proof*

We bound the term $\left( \frac{1}{H} u_{h,t} , u_h^D \right)$, with Cauchy–Schwarz, Poincare–Friedrichs (19), and weighted Young’s inequality with $\delta = \frac{\epsilon }{\sqrt{\beta }}$:

38 $$\begin{aligned} \left( \frac{1}{H} u_{h,t}^D , u_h^D \right)\le & {} \frac{C_P}{2\sqrt{H_*}} \left[ \frac{\epsilon }{\sqrt{\beta }} \left\| u_{h,t} \right\| _{\frac{1}{H}}^2 + \frac{\sqrt{\beta }}{\epsilon } \left\| \nabla \cdot u_h^D \right\| ^2 \right] \nonumber \\= & {} \frac{C_P\epsilon }{\sqrt{H_*\beta }} \left[ \frac{1}{2} \left\| u_{h,t} \right\| _{\frac{1}{H}}^2 + \frac{\beta }{2\epsilon ^2} \left\| \nabla \cdot u_h^D \right\| ^2 \right] \nonumber \\= & {} \frac{C_P\epsilon }{\sqrt{H_*\beta }} E(t). \end{aligned}$$

So, then, we have

39 $$\begin{aligned} A(t)\le & {} \left( 1 + \frac{\alpha C_P \epsilon }{\sqrt{\beta H_*}} \right) E(t), \nonumber \\ A(t)\ge & {} \left( 1 - \frac{\alpha C_P \epsilon }{\sqrt{\beta H_*}} \right) E(t), \end{aligned}$$

and the result follows thanks to the assumption (36).

Showing that *B*(*t*) is bounded above by a constant times *E*(*t*) is straightforward, but not needed for our damping results.

### **Lemma 2**

Suppose that

40 $$\begin{aligned} 0 < \alpha \le \alpha _2 \equiv \frac{2 C_*}{1 + \chi }, \end{aligned}$$

where

41 $$\begin{aligned} \chi = \left( 2 + \frac{C_P^2 \left( 1 + \epsilon C^* \right) ^2}{ \beta H_*} \right) . \end{aligned}$$

Then

42 $$\begin{aligned} B(t) \ge \alpha E(t). \end{aligned}$$

### *Proof*

We use Cauchy–Schwarz, the bounds $0 < C_* \le C \le C^*$ and $|f| \le 1$, and Young’s inequality with weight $\delta > 0$ to write

43 $$\begin{aligned} B(t)\ge & {} \left( C_* - \alpha \right) \left\| u_{h,t} \right\| _{\frac{1}{H}}^2 + \frac{\alpha \beta }{\epsilon ^2} \left\| \nabla \cdot u_h^D \right\| ^2 \nonumber \\&- \frac{\alpha C_P}{\epsilon \sqrt{H_*}} \left( C^* \epsilon + 1 \right) \left\| u_{h,t} \right\| _{\frac{1}{H}} \left\| \nabla \cdot u_h^D \right\| \\\ge & {} \left[ 2 C_* - \alpha \left( 2 + \frac{C_P \left( 1 + \epsilon C^* \right) }{\epsilon \sqrt{H_*} \delta } \right) \right] \frac{1}{2} \left\| u_{h,t} \right\| _{\frac{1}{H}}^2 \\&+\,\, \alpha \left[ 2 - \frac{\epsilon C_P \left( 1 + \epsilon C^* \right) }{\beta \sqrt{H_*}} \delta \right] \frac{\beta }{2\epsilon ^2} \left\| \nabla \cdot u_h^D \right\| ^2. \end{aligned}$$

Next, it remains to select $\delta$ and $\alpha$ to make the coefficients of each norm positive and also balance the terms. First, we pick

$$\begin{aligned} \delta = \frac{\beta \sqrt{H_*}}{\epsilon C_P \left( 1 + \epsilon C^* \right) }, \end{aligned}$$

and calculating that

$$\begin{aligned} \frac{C_P \left( 1 + \epsilon C^* \right) }{\epsilon \sqrt{H_*} \delta } = \frac{C_P^2 \left( 1 + \epsilon C^* \right) ^2}{ \beta H_*}, \end{aligned}$$

we have that

44 $$\begin{aligned} B(t)\ge & {} \left[ 2 C_* - \alpha \left( 2 + \frac{C_P^2 \left( 1 + \epsilon C^* \right) ^2}{ \beta H_*} \right) \right] \frac{1}{2} \left\| u_{h,t} \right\| _{\frac{1}{H}}^2 + \alpha \frac{\beta }{2\epsilon ^2} \left\| \nabla \cdot u_h^D \right\| ^2 \nonumber \\= & {} \left( 2 C_* - \alpha \chi \right) \frac{1}{2} \left\| u_{h,t} \right\| _{\frac{1}{H}}^2 + \frac{\alpha \beta }{2\epsilon ^2} \left\| \nabla \cdot u_h^D \right\| ^2. \end{aligned}$$

We let $\alpha _2$ be the solution to

$$\begin{aligned} 2 c_* - \alpha _2 \chi = \alpha _2, \end{aligned}$$

so that

45 $$\begin{aligned} \alpha _2 \equiv \frac{2 C_*}{1 + \chi }. \end{aligned}$$

If we pick $\alpha = \alpha _2$, then we have the lower bound for *B*(*t*) is exactly $\alpha E(t)$. However, we are also constrained to pick $\alpha \le \min \{\alpha _1,\alpha _2\}$ in order to guarantee that the lower bounds for *A*(*t*) is positive as well. If we have $\alpha \le \alpha _2$, then

$$\begin{aligned} 2 C_* - \alpha \chi \ge 2 C_* - \alpha _2 \chi = \alpha _2 \ge \alpha , \end{aligned}$$

and so we also have

46 $$\begin{aligned} B(t) \ge \alpha E(t). \end{aligned}$$

We combine these two lemmas to give our exponential damping result.

### **Theorem 1**

Let $\alpha _1$ and $\alpha _2$ be defined by (36) and (40), respectively. Then, for any $0 < \alpha \le \min \{ \alpha _1,\alpha _2 \}$, and any $t > 0$, we have

47 $$\begin{aligned} E(t) \le 3 E(0) e^{-\frac{2\alpha }{3} t}. \end{aligned}$$

### *Proof*

In light of (33), (42), and the lower bound in (37), we have that

48 $$\begin{aligned} A^\prime (t) + \frac{2\alpha }{3} A(t) \le 0, \end{aligned}$$

so that

49 $$\begin{aligned} A(t) \le A(0) e^{-\frac{2\alpha }{3} t}. \end{aligned}$$

Using the upper and lower bounds of *A* in (37) gives the desired estimate.

This result shows that the damping term drives an unforced system to one with a steady, solenoidal velocity field, in which the Coriolis force balances the pressure gradient term, i.e. in a state of geostrophic balance. Using the second equation in (5), we also know that the linearized height disturbance is steady in time in this case. These facts together lead to an elliptic equation for the steady state

50 $$\begin{aligned} \begin{aligned}&\displaystyle \left( \frac{C}{H} u_h , v_h \right) + \frac{1}{\epsilon } \left( \frac{f}{H} u_h^\perp , v_h \right) - \frac{\beta }{\epsilon ^2} \left( \eta _h , \nabla \cdot v_h \right) = 0 \\& \displaystyle \left( \nabla \cdot u_h, w_h \right) = 0 \end{aligned} \end{aligned}$$

It is easy to see that this problem is coercive on the divergence-free subspaces and thus is well-posed. Hence, with zero forcing, both $u_h$ and $\eta _h$ equal zero is the only solution. The zero-energy steady state then cannot have a nonzero solenoidal part. Moreover, the exponentially decay of $\Vert u_t \Vert$ toward zero forces *u* to reach its steady state quickly, driving both $u^D$ and $u^S$ toward zero at an exponential rate. Finally, since $\eta _t = -\nabla \cdot u$ almost everywhere, the exponential damping of $\Vert \nabla \cdot u \Vert$ also forces $\eta$ toward its zero steady state at the same rate.

Now, we turn to the case where the forcing term is nonzero, adapting this damping result to give long-time stability. The same techniques as before now lead to

51 $$\begin{aligned} A^\prime (t) + B(t) = \left( \widetilde{F} , u_{h,t} + \alpha u_h^D \right) . \end{aligned}$$

### **Theorem 2**

For any $0 < \alpha \le \min \{ \alpha _1,\alpha _2\}$ and

52 $$\begin{aligned} K_\alpha \equiv \frac{1}{2} \left[ 1 + \frac{\alpha ^2 C_P^2\epsilon ^2}{\beta H_*^2} \right] , \end{aligned}$$

we have the bound

53 $$\begin{aligned} E(t) \le 3 e^{-\frac{\alpha }{3}t} E(0) + \frac{K_\alpha }{\alpha } \int _0^t e^{\frac{\alpha }{3} \left( s - t \right) } \left\| \widetilde{F} \right\| _{H}^2 ds. \end{aligned}$$

### *Proof*

We bound the right-hand side of (51) by

54 $$\begin{aligned} \left( \widetilde{F} , u_{h,t} + \alpha u_h^D \right)\le & {} \left\| \widetilde{F} \right\| _{H} \left\| u_{h,t} \right\| _{\frac{1}{H}} + \alpha C_P \left\| \widetilde{F} \right\| _{H} \left\| \nabla \cdot u_h^D \right\| _{\frac{1}{H}} \nonumber \\\le & {} \left[ \frac{H^*}{2 \delta _1} + \frac{\alpha C_P}{2 \delta _2} \right] \left\| \widetilde{F} \right\| _{H}^2 + \frac{\delta _1}{2} \left\| u_{h,t} \right\| _{\frac{1}{H}} + \frac{\alpha C_P \delta _2}{2 H_*}\left\| \nabla \cdot u_h^D \right\| ^2\nonumber \\ \end{aligned}$$

We put $\delta _2 = \frac{\beta \delta _1 H_*}{\alpha C_P \epsilon ^2}$ to find

55 $$\begin{aligned} \left( \widetilde{F} , u_{h,t} + \alpha u_h^D \right) \le \frac{1}{\delta _1} K_\alpha \left\| \widetilde{F} \right\| _{H}^2 + \delta _1 E(t). \end{aligned}$$

This turns (51) into the differential inequality

56 $$\begin{aligned} A^\prime (t) + B(t) \le \frac{K_\alpha }{\delta _1} \left\| \widetilde{F} \right\| _{H}^2 + \delta _1 E(t). \end{aligned}$$

Using  (42), we obtain

57 $$\begin{aligned} A^\prime (t) + \alpha E(t) \le \frac{K_\alpha }{\delta _1} \left\| \widetilde{F} \right\| _{H}^2 + \delta _1 E(t). \end{aligned}$$

At this point, we specify $\delta _1 = \frac{\alpha }{2}$ so that, with (37) we have

58 $$\begin{aligned} A^\prime (t) + \frac{\alpha }{3} A(t) \le \frac{K_\alpha }{\alpha } \left\| \widetilde{F} \right\| _{H}^2. \end{aligned}$$

This leads to the bound on *A*(*t*)

59 $$\begin{aligned} A(t) \le e^{-\frac{\alpha }{3} t} A(0) + \frac{K_\alpha }{\alpha } \int _0^t e^{\frac{\alpha }{3}\left( s-t\right) } \left\| \widetilde{F} \right\| _{H}^2 ds. \end{aligned}$$

Using (37) again gives the desired result.

These stability results have important implications for tidal computations. Theorem 2 shows long-time stability of the system. Our stability result also shows that the semidiscrete method captures the three-way geotryptic balance between Coriolis, pressure gradients, and forcing. Moreover, we also can demonstrate that “spin-up”, the process by which in practice tide models are started from an arbitrary initial condition and run until they approach their long-term behavior, is justified for this method. To see this, the difference between any two solutions with equal forcing but differing initial conditions will satisfy the same (6) with nonzero initial conditions and zero forcing. Consequently, the difference must approach zero exponentially fast. This means that we can define a global attracting solution in the standard way [that is, take $\eta (x,t;t^*)$, $u(x,t;t^*)$ for $0 > t^*$ and $t > t^*$ as the solution starting from zero initial conditions at $t^*$ and define the global attracting solution as the limit as $t^* \rightarrow -\infty$], to which the solution for any condition becomes exponentially close in finite time. The error estimates we demonstrate in the next section then can be used to show that the semidiscrete finite element solution for given initial conditions approximates this global attracting solution arbitrarily well by picking *t* large enough that the difference between the exact solution with those initial conditions and the global attracting solution is small and then letting *h* be small enough that the finite element solution approximates that exact solution well.

## Error estimates

Optimal a priori error estimates follow by applying our stability estimates to a discrete equation for the difference between the numerical solution and a projection of the true solution. We define

60 $$\begin{aligned} \chi\equiv & {} \Pi u - u, \nonumber \\ \rho\equiv & {} \pi \eta - \eta , \nonumber \\ \theta _h\equiv & {} \Pi u - u_h, \nonumber \\ \zeta _h\equiv & {} \pi \eta - \eta _h. \end{aligned}$$

The projections $\Pi u$ and $\pi \eta$ satisfy the first-order system

61 $$\begin{aligned} \begin{aligned}&\displaystyle \left( \frac{1}{H} \Pi u_{t} , v_h \right) + \frac{1}{\epsilon } \left( \frac{f}{H} \Pi u^\perp , v_h \right) - \frac{\beta }{\epsilon ^2} \left( \pi \eta , \nabla \cdot v_h \right) + \left( \frac{C}{H} \Pi u , v_h \right) \\&\displaystyle \quad = \left( F + \frac{f}{\epsilon H} \chi + \frac{1}{H}\chi _t +\frac{C}{H} \chi , v_h \right) ,\\&\displaystyle \quad \left( \pi \eta _{t} , w_h \right) + \left( \nabla \cdot \Pi u , w_h \right) = 0. \end{aligned} \end{aligned}$$

Subtracting the discrete equation (5) from this gives

62 $$\begin{aligned} \begin{aligned}&\left( \frac{1}{H} \theta _{h,t} , v_h \right) + \frac{1}{\epsilon } \left( \frac{f}{H} \theta _h^\perp , v_h \right) - \frac{\beta }{\epsilon ^2} \left( \zeta _h , \nabla \cdot v_h \right) + \left( \frac{C}{H} \theta _h , v_h \right) \\&\quad = \left( \frac{f}{\epsilon H} \chi + \frac{1}{H} \chi _t + \frac{C}{H} \chi , w_h \right) , \\&\left( \zeta _{h,t} , w_h \right) + \left( \nabla \cdot \theta _h , w_h \right) = 0. \end{aligned} \end{aligned}$$

By choosing the initial conditions for the discrete problem as $u_h(\cdot ,0) = \Pi u_0$ and $\eta _h(\cdot ,0) = \pi \eta _0$, the initial conditions for these error equations are

63 $$\begin{aligned} \begin{aligned}&\theta _h(\cdot ,0) = 0,\\&\eta _h(\cdot ,0) = 0. \end{aligned} \end{aligned}$$

We start with $L^2$ estimates for the height and momentum variables, based on the stability result for the first order system.

### **Proposition 4**

For any $t > 0$, provided that $u,u_t \in L^2([0,t],H^{k+\sigma }({\varOmega }))$,

64 $$\begin{aligned}&\frac{1}{2} \left\| \theta _h\left( \cdot , t \right) \right\| _{\frac{1}{H}}^2 + \frac{\beta }{2\epsilon ^2} \left\| \zeta _h\left( \cdot , t \right) \right\| ^2 \nonumber \\&\quad \le \frac{C_\pi ^2 h^{2\left( k + \sigma \right) }}{C_* H_*} \int _0^t \frac{1}{\epsilon } \left| u\left( \cdot , s \right) \right| _{k+\sigma }^2 + \left| u_t\left( \cdot , s \right) \right| _{k+\sigma }^2 + C^* \left| u\left( \cdot , s \right) \right| _{k+\sigma }^2 ds. \end{aligned}$$

### *Proof*

We apply Proposition 3 to (62) to find

65 $$\begin{aligned} \frac{1}{2} \left\| \theta _h\left( \cdot , t \right) \right\| _{\frac{1}{H}}^2 + \frac{\beta }{2\epsilon ^2} \left\| \zeta _h\left( \cdot , t \right) \right\| ^2 \le \frac{1}{2C_*} \int _0^t \left\| \frac{f}{\epsilon H} \chi + \frac{1}{H} \chi _t + \frac{C}{H} \chi \left( \cdot , s \right) \right\| _{H}^2 ds. \end{aligned}$$

Note that for any *g*,

$$\begin{aligned} \left\| \frac{1}{H} g \right\| _{H}^2 = \int _{\varOmega } H \left( \frac{1}{H} \left| g \right| \right) ^2 dx = \int _{\varOmega } \frac{1}{H} \left| g \right| ^2 dx = \left\| g \right\| _{\frac{1}{H}}^2. \end{aligned}$$

Using this, that $(a+b)^2 \le 2 \left( a^2 + b^2 \right)$, and norm equivalence bounds the right-hand side above by

$$\begin{aligned}&\frac{1}{C_*H_*} \int _0^t \left\| \frac{f}{\epsilon } \chi \left( \cdot , s \right) \right\| ^2 + \left\| \chi _t\left( \cdot , s \right) \right\| ^2 + \left\| C \chi \left( \cdot , s \right) \right\| ^2 ds \\&\quad \le \frac{1}{C_*H_*} \int _0^t \frac{1}{\epsilon } \left\| \chi \left( \cdot , s \right) \right\| ^2 + \left\| \chi _t\left( \cdot , s \right) \right\| ^2 + C^* \left\| \chi \left( \cdot , s \right) \right\| ^2 ds \end{aligned}$$

and the approximation estimate (14) finishes the proof.

Since

$$\begin{aligned} \frac{1}{2} \left\| \left( u - u_h \right) \right\| _{\frac{1}{H}}^2 + \frac{\beta }{2 \epsilon ^2} \left\| \eta - \eta _h \right\| ^2 \le \left\| \rho \right\| _{\frac{1}{H}}^2 + \left\| \zeta _h \right\| _{\frac{1}{H}}^2 + \frac{\beta }{2 \epsilon ^2} \left\| \chi \right\| ^2 + \frac{\beta }{2 \epsilon ^2} \left\| \theta \right\| ^2, \end{aligned}$$

we combine this result with the approximation estimates to obtain

### **Theorem 3**

If the above hypotheses hold, and also $u \in L^\infty ([0,t];H^{k+\sigma }({\varOmega }))$ and $\eta \in L^\infty ([0,t];H^k({\varOmega }))$, we have the error estimate

66 $$\begin{aligned}&\frac{1}{2} \left\| \left( u - u_h \right) \left( \cdot , t \right) \right\| _{\frac{1}{H}}^2 + \frac{\beta }{2 \epsilon ^2} \left\| \left( \eta - \eta _h \right) \left( \cdot , t \right) \right\| ^2\nonumber \\&\quad \le \frac{C_\Pi ^2 h^{2\left( k + \sigma \right) }}{H_*} \left| u \left( \cdot , t \right) \right| _{ k + \sigma }^2 + \frac{C_\pi ^2 \beta h^{2k}}{\epsilon ^2} \left| \eta \left( \cdot , t \right) \right| _{k}^2 \nonumber \\&\qquad +\,\, \frac{2 C_\pi ^2 h^{2\left( k + \sigma \right) }}{C_* H_*} \int _0^t \left| u_t\left( \cdot , s \right) \right| _{k+\sigma }^2 + C^* \left| u\left( \cdot , s \right) \right| _{k+\sigma }^2 ds. \end{aligned}$$

Note that our bound on the error equations in Proposition 4 depends only on the approximation properties of the velocity space, while the full error in the finite element solution depends on the approximation properties of both spaces. Consequently, the velocity approximation using BDM elements is suboptimal. Using RT or BDFM elements, both fields are approximated to optimal order.

Now, we use our estimates based on the second-order system to obtain error estimates for the time derivative and divergence of the momentum. The projection $\Pi u$ satisfies the perturbed equation

67 $$\begin{aligned}&\left( \frac{1}{H} \Pi u_{tt} , v_h \right) + \frac{1}{\epsilon } \left( \frac{f}{H} \Pi u_{t}^\perp , v_h \right) + \frac{\beta }{\epsilon ^2} \left( \nabla \cdot \Pi u , \nabla \cdot v_h \right) + \left( \frac{C}{H} \Pi u_{t} , v_h \right) \nonumber \\&\quad = \left( \frac{1}{H} \chi _{tt} , v_h \right) + \frac{1}{\epsilon } \left( \frac{1}{H} \chi ^\perp _t , v_h \right) + \left( \frac{C}{H} \chi _t , v_h \right) + \left( \widetilde{F} , v_h \right) . \end{aligned}$$

As in the first-order case, we have $\theta _h \equiv \Pi u - u_h$, and subtracting (6) from (67) gives

68 $$\begin{aligned}&\left( \frac{1}{H} \theta _{h,tt} , v_h \right) + \frac{1}{\epsilon } \left( \frac{f}{H} \theta _{h,t}^\perp , v_h \right) + \frac{\beta }{\epsilon ^2} \left( \nabla \cdot \theta _{h} , \nabla \cdot v_h \right) + \left( \frac{C}{H} \theta _{h,t} , v_h \right) \nonumber \\&\quad = \left( \frac{1}{H} \chi _{tt} , v_h \right) + \frac{1}{\epsilon } \left( \frac{f}{H} \chi ^\perp _t , v_h \right) + \left( \frac{C}{H} \chi _t , v_h \right) . \end{aligned}$$

Theorem 2 and approximation estimates for $\chi$ give this result.

### **Proposition 5**

Let $\alpha = \alpha _* = \min \{ \alpha _1 , \alpha _2\}$ and suppose that $u_t, u_{tt} \in L^1(0,T;H_{k+1})$. Then

69 $$\begin{aligned}&\frac{1}{2} \left\| \theta _{h,t} \right\| _{\frac{1}{H}}^2 + \frac{\beta }{2\epsilon ^2} \left\| \nabla \cdot \theta _h \right\| ^2\nonumber \\&\quad \le \frac{K_{\alpha _*}C_\Pi ^2 h^{2\left( k+\sigma \right) }}{\alpha _* H_*} \int _0^t e^{\frac{\alpha _*}{3}\left( s - t \right) } \left( \left| u_{tt} \right| _{k+1}^2 + \left( \frac{1}{\epsilon } + C^* \right) \left| u_{t} \right| _{k+1}^2 \right) . \end{aligned}$$

### *Proof*

Applying the stability estimate to (68), noting that $\theta _h =0$ at $t=0$ gives

70 $$\begin{aligned} \frac{1}{2} \left\| \theta _{h,t} \right\| _{\frac{1}{H}}^2 + \frac{\beta }{2\epsilon ^2} \left\| \nabla \cdot \theta _h \right\| ^2 \le \frac{K_{\alpha _*}}{\alpha _*} \int _0^t e^{-\frac{\alpha _*}{3}\left( s - t \right) } \left( \left\| \xi _{tt} \right\| _{\frac{1}{H}}^2 + \left( \frac{1}{\epsilon } + C^* \right) \left\| \xi _{t} \right\| _{\frac{1}{H}}^2 \right) ,\nonumber \\ \end{aligned}$$

and applying the norm equivalence and approximation estimate (16) gives the result.

It is straightforward to get from here to a bound on the error

71 $$\begin{aligned} \varepsilon ^2 \equiv \frac{1}{2} \left\| \left( u_t - u_{h,t} \right) \left( \cdot , t \right) \right\| _{\frac{1}{H}}^2 + \frac{\beta }{2 \epsilon ^2} \left\| \nabla \cdot \left( u - u_h \right) \left( \cdot , t \right) \right\| ^2. \end{aligned}$$

### **Theorem 4**

If the above assumptions hold, and also $u_t,u_{tt} \in L^\infty ([0,t];H^{k+1}({\varOmega }))$, then

72 $$\begin{aligned} \varepsilon ^2\le & {} \frac{C_\Pi ^2 h^{2\left( k + \sigma \right) }}{H_*} \left| u_t \left( \cdot , t \right) \right| _{ k + \sigma }^2 + \frac{C_\pi ^2 \beta h^{2k}}{\epsilon ^2} \left| u \left( \cdot , t \right) \right| _{k+1}^2 \nonumber \\&+ \frac{2K_{\alpha _*}C_\Pi ^2 h^{2\left( k+\sigma \right) }}{\alpha _* H_*} \int _0^t e^{-\frac{\alpha _*}{3}\left( s - t \right) } \left( \left| u_{tt} \right| _{k+1}^2 + \left( \frac{1}{\epsilon } + C^* \right) \left| u_{t} \right| _{k+1}^2 \right) . \end{aligned}$$

## Numerical results

In this section we present some numerical experiments that illustrate the estimates derived in the previous sections. In all cases the equations are discretized in time using the implicit midpoint rule. The domain is the unit sphere, centred on the origin, which is approximated using triangular elements arranged in an icosahedral mesh structure (see Appendix A for extensions of the results of this paper to embedded surfaces such as the sphere). All numerical results are obtained using the open source finite element library, Firedrake (http://www.firedrake.org).

**Fig. 1:**
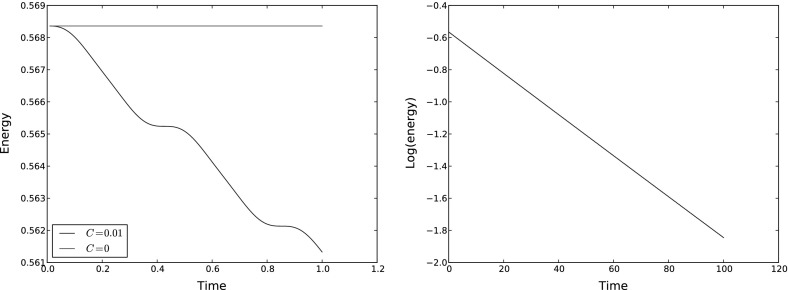
Plots of the evolution of energy with time in the cases $C=0$ and $C=0.01$. *Left* energy-time plots for $C=0$ and $C=0.01$, over the time interval $0<t<1$. For $C=0$ we observe exact energy conservation as expected. For $C=0.01$ the energy is monotonically decreasing as expected. *Right* energy-time plot for $C=0.01$ on a logarithmic scale over the time interval $0<t<50$. Then energy is decaying exponentially in time, as expected

First, we verify the energy behavior in the absence of dissipation, i.e. $C=0$. The variables were initialized with ${u}=0$ and $\eta =xyz$, and the equations were solved with parameters $\epsilon =\beta =0.1$, $f=1$, $H=1 + 0.1\exp (-x^2)$, and ${\varDelta } t=0.01$. The energy is conserved by the continuous-time spatial semi-discretization, and is quadratic. Since the implicit midpoint rule time-discretization preserves all quadratic invariants (see [24], for example), we expect exact energy conservation in this case; this was indeed observed as shown in Fig. 1. Upon introducing a positive dissipation constant $C=0.1$, we observe both that the energy is monotonically decreasing (as implied by Proposition 2), and is scaling exponentially in time (as implied by Theorem 1). These results are also illustrated in Fig. 1.

**Fig. 2:**
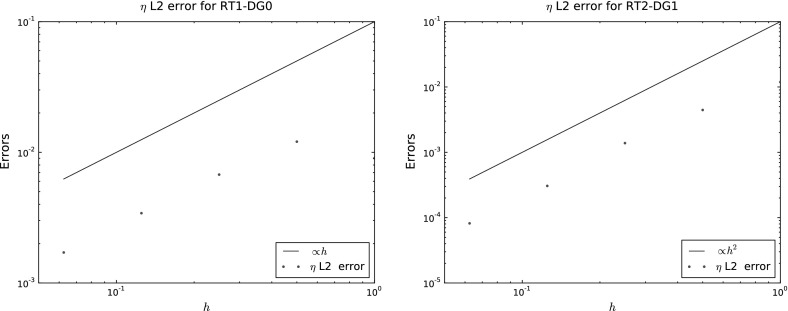
Convergence plots obtained from the method of manufactured solutions, showing the time-integrated $L^2$ error in $\eta$ against the typical element edge length *h*. *Left* plot for RT1-DG0, the error is proportional to *h* as expected. *Right* plot for RT2-DG1, the error is proportional to $h^2$ as expected

Second, we verify the convergence results proved in Sect. 5. This was done by constructing a reference solution using the method of manufactured solutions, i.e. by choosing the solution

$$\begin{aligned} u= & {} \cos ({\varOmega } t)\left( -\frac{1}{12}(yz(1 - 3x^2), -\frac{1}{12}(xz(1 - 3y^2), -\frac{1}{12}(xy(1 - 3z^2) \right) , \\ \eta= & {} -\sin ({\varOmega } t)\frac{xyz}{12}, \end{aligned}$$

where we have expressed the velocity in three dimensional coordinates even though it is constrained to remain tangential to the sphere. Here $\eta$ and *u* are chosen to solve the continuity equation for $\eta$ exactly, and *F* is then chosen so that the *u* equation is satisfied. We used the parameters $\epsilon =\beta =0.1$, $f=H=1$, $C=1000$, ${\varOmega }=2$, and chose ${\varDelta } t=10^{-5}$ in order to isolate the error due to spatial discretization only. We ran the solutions until $t=0.3$ and computed the time-averaged $L^2$ error for $\eta$. Plots are shown in Fig. 2; they confirm the expected first order convergence rate for $V=\hbox {RT1}$, $Q=$ DG0, and the expected second order convergence rate for $V=\hbox {RT2}$, $Q= \hbox {DG1}$.

**Fig. 3:**
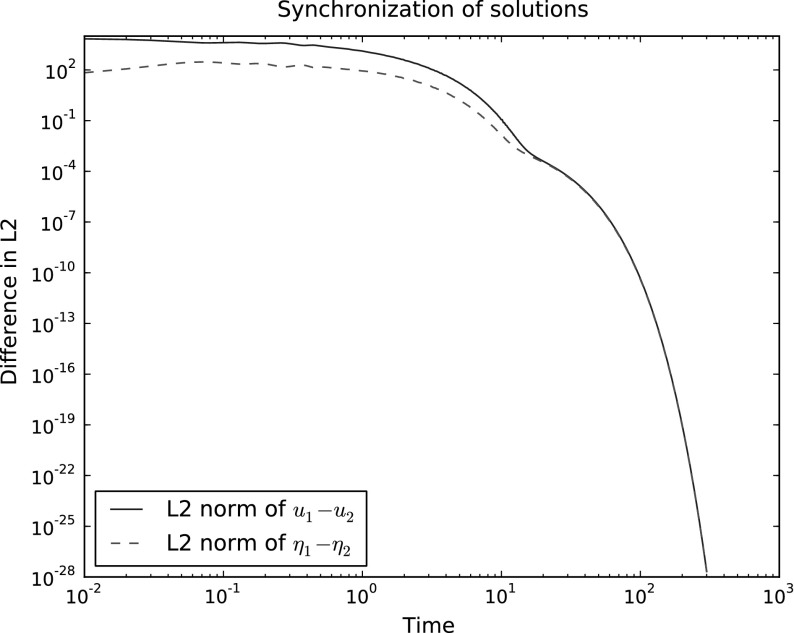
Plot of the $L^2$ difference between two pairs of solutions $(u_1,\eta _1)$ and $(u_2,\eta _2)$ with different randomly generated initial conditions but the same forcing, as a function of time. As expected, the difference converges to zero (eventually with exponential rate) as $t\rightarrow \infty$, demonstrating the absence of spurious solutions

Finally, we illustrate that this type of discretization excludes the possibility of spurious attracting solutions. In the case of the linear forced-dissipative tidal equations with time-dependent forcing, the continuous equations have the property that the solutions lose memory of the initial conditions exponentially quickly with timescale determined from *C* and the other parameters (and bounded by $\alpha$ in Theorem 1). As discussed among our stability results, any two solutions with different initial conditions should converge to the same solution as $t\rightarrow \infty$. We illustrate this by randomly generating initial conditions for two solutions $(u_1,\eta _1)$ and $(u_2,\eta _2)$ with the same time-periodic forcing,

$$\begin{aligned} (F,v) = \frac{\beta }{\epsilon ^2} \sin (t) (xyz,\nabla \cdot v), \quad \forall v \in V, \end{aligned}$$

and measuring the difference between them as $t\rightarrow \infty$. In performing this test, care must be taken to ensure that $\eta _1$ and $\eta _2$ both have zero mean as implied by the perturbative derivation of the linear equations (since the dissipation cannot influence the mean component). In this experiment, we used the parameters $\epsilon =\beta = 0.1$, $C = 10.0$, ${\varDelta } t=0.01$ and we used an icosahedral mesh of the sphere at the fourth level of refinement. We indeed observed that the two solutions converge to each other exponentially quickly in the $L^2$ norm, as illustrated in Fig. 3.

## Conclusions and future work

We have presented and analyzed mixed finite element methods for the linearized rotating shallow equations with forcing and linear drag terms. Our more delicate energy estimates rely on an equivalence between the first order form and a second order form, and this equivalence itself relies on fundamental properties of classical $H(\mathrm {div})$ finite elements. In particular, our estimates show that the mixed spatial discretization accurately captures the long-term energy of the system, in which damping balances out forcing to prevent energy accumulation. Because of the linearity of the problem, our energy estimates also give rise to a priori error estimates that are optimal for Raviart–Thomas and Brezzi–Douglas–Fortin–Marini elements. Numerical results confirm both the stability and convergence theory given.

In the future, we hope to extend this work in several directions. First, we hope to study the more realistic quadratic damping model, which will require new techniques to handle the nonlinearity. Second, our estimates have only handled the semidiscrete case, and it is well-known that time-stepping schemes do not always preserve the right energy balances. Without damping or forcing, the implicit midpoint method preserves exact energy balance, and a symplectic Euler method will exactly conserve an approximate functional for linear problems. It remains to be seen how to give a rigorous fully discrete analysis, either including damping by a fractional step or fully implicit method. Finally, even explicit or symplectic time-stepping will require us to consider linear algebraic problems, as it is typically not possible to perform mass lumping for $H(\mathrm {div})$ spaces on triangular meshes. Implicit methods will require additional care.

## Appendix A: Extension to the sphere and other embedded manifolds

Global tidal simulations are performed in spherical geometry, so it is necessary to consider mixed finite element discretization using meshes of isoparametric elements that approximate the sphere. This constitutes a variational crime since the domain $M_h$ supporting the mesh is only the same as the manifold *M* in the limit $h\rightarrow 0$. Recently, the topic of mixed finite elements on embedded manifolds was comprehensively analyzed by [15], following previous work on nodal finite elements. Here, we sketch out how to use their approach to extend the results of this paper to embedded manifolds.

In the case of curved domains such as the surface of the sphere, $H(\mathrm {div})$ elements are implemented *via* Piola transforms from a reference triangle. This means that (a) the velocity fields are always tangential to the mesh element, and (b) normal fluxes $u\cdot n$ take the same value on each side of element boundaries, as required to obtain a divergence that is bounded in $L^2$ (an approach to practical implementation of these finite element spaces on manifolds is provided by [29]). Similarly, the discontinuous $L^2$ spaces are implemented using a transformation from the reference triangle that includes scaling by the determinant of the Jacobian $J_e$; this ensures that the surface divergence maps from $V_h$ onto $W_h$.

In this case $V_h\not \subset V$, $W_h \not \subset W$. [15] dealt with this problem by constructing operators $\iota _{V_h}:V_h \rightarrow V$ and $\iota _{W_h}:W_h\rightarrow W$ such that

$$\begin{aligned} \Pi \circ \iota _{V_h}= {{\mathrm{Id}}}_{V_h}, \quad \pi \circ \iota _{W_h} = {{\mathrm{Id}}}_{W_h}, \end{aligned}$$

where $\Pi$ and $\pi$ are projections from *V* to $V_h$ and *W* to $W_h$ respectively; these two operators commute with $\nabla \cdot$ defined on $M_h$. In particular,

$$\begin{aligned} \left( \pi \eta ,w_h\right) = \left( \eta ,\iota _{W_h}w_h \right) , \quad \forall w_h \in W_h, \, \eta \in W. \end{aligned}$$

The estimates (14–16) then hold with $\iota _{V_h}\circ \Pi$ and $\iota _{W_h}\circ \pi$ replacing $\Pi$ and $\pi$ respectively, provided that the polynomial expansion of the element geometries in $M_h$ have at the same approximation order as $V_h$ and $W_h$. There is also still a discrete Poincaré–Friedrichs inequality for $V_h$. This means that all of our stability results from Sect. 4 hold in the manifold case, and it remains to deal with the error estimates. This is done by introducing further variables $u_h'\in V_h$, $\eta '_h\in W_h$ satisfying

73 $$\begin{aligned}&\left( \frac{1}{H} \iota _{V_h}u_{h,t}' , \iota _{V_h} v_h \right) + \frac{1}{\epsilon } \left( \frac{f}{H} (\iota _{V_h}u_h')^\perp , \iota _{V_h} v_h \right) \nonumber \\&\quad \quad - \frac{\beta }{\epsilon ^2} \left( \iota _{W_h}\eta '_h , \iota _{W_h}\nabla \cdot v_h \right) + \left( \frac{C}{H} \iota _{V_h}u'_h , \iota _{V_h}v_h \right) , \nonumber \\&\quad = \left( F, \iota _{V_h}v_h \right) \left( \eta '_{h,t} , w_h \right) + \left( \nabla \cdot u_h' ,w_h \right) = 0. \end{aligned}$$

This equation is of the form (5) but with a modified inner product on $V_h$. Therefore, all of our stability estimates also hold for this modified equation.

We split the error in *u* and $\eta$ by writing

74 $$\begin{aligned} \begin{aligned} u - \iota _{V_h}u_h&= -\chi + \iota _{V_h}\theta '_h + \iota _{V_h}\theta _h,\\ \eta - \iota _{W_h}\eta _h&= -\rho + \iota _{W_h}\zeta '_h + \iota _{W_h}\zeta _h, \end{aligned} \end{aligned}$$

where

75 $$\begin{aligned} \begin{aligned} \chi&\equiv \iota _{V_h}\Pi u - u, \\ \rho&\equiv \iota _{W_h}\pi \eta - \eta , \\ \theta '_h&\equiv \Pi u - u_h', \\ \zeta '_h&\equiv \pi \eta _ - \eta _h'.\\ \theta _h&\equiv u_h' - u_h, \\ \zeta _h&\equiv \eta _h' - \eta _h. \end{aligned} \end{aligned}$$

We can bound $\theta '_h$ and $\zeta '_h$ by applying Proposition 3 adapted to Eq. (73), i.e. by substituting $v=\iota _{V_h}v_h$ into (4) and rearranging so that it takes the form of (73) with a forcing defined in terms of *u*, then subtracting (73). Similarly, $\theta _h$ and $\zeta _h$ may be bounded by rearranging Eq. (73) into the form of (4), then subtracting (4). Terms appear that are proportional to $\Vert {{\mathrm{Id}}}-J\Vert$ where

$$\begin{aligned} J_{V_h} = \iota _{V_h}^*\iota _{V_h}, \quad J_{W_h} = \iota _{W_h}^*\iota _{W_h}, \end{aligned}$$

and $\Vert {{\mathrm{Id}}}-J\Vert$ is the maximum of the operator norms of ${{\mathrm{Id}}}_{V_h}-J_{V_h}$ and ${{\mathrm{Id}}}_{W_h}-J_{W_h}$. [15] showed that $\Vert {{\mathrm{Id}}}-J\Vert$ converges to zero as $h\rightarrow 0$ with rate determined by the order of polynomial approximation in the isoparametric mapping. Hence we obtain a manifold version of Theorem 3, with $u_h$ and $\eta _h$ substituted by $\iota _{V_h}u_h$ and $\iota _{W_h}\eta _h$ respectively. Similar techniques lead to a manifold version of Theorem 4.

## References

[1] Arnold DN, Falk RS, Winther R (2006). Finite element exterior calculus, homological techniques, and applications. Acta Numer..

[2] Arnold DN, Falk RS, Winther R (2010). Finite element exterior calculus: from Hodge theory to numerical stability. Bull. Am. Math. Soc..

[3] Brezzi F, Fortin M (1991). Mixed and Hybrid Finite Element Methods.

[4] Brezzi F, Douglas J, Marini LD (1985). Two families of mixed finite elements for second order elliptic problems. Numer. Math..

[5] Comblen R, Lambrechts J, Remacle JF, Legat V (2010). Practical evaluation of five partly discontinuous finite element pairs for the non-conservative shallow water equations. Int. J. Numer. Methods Fluids.

[6] Cotter C, Ham D (2011). Numerical wave propagation for the triangular P1DG-P2 finite element pair.. J. Comput. Phys..

[7] Cotter CJ, Shipton J (2012). Mixed finite elements for numerical weather prediction. J. Comput. Phys..

[8] Cotter CJ, Thuburn J (2014). A finite element exterior calculus framework for the rotating shallow-water equations. J. Comput. Phys..

[9] Cowsar LC, Dupont TF, Wheeler MF (1990). A priori estimates for mixed finite element methods for the wave equation. Comput. Methods Appl. Mech. Eng..

[10] Danilov S (2010). On utility of triangular C-grid type discretization for numerical modeling of large-scale ocean flows. Ocean Dynam..

[11] Foreman, M., Henry, R., Walters, R., Ballantyne, V.: A finite element model for tides and resonance along the north coast of British Columbia. J. Geophys. Res.: Oceans (1978–2012) **98**(C2), 2509–2531 (1993)

[12] Garrett C, Kunze E (2007). Internal tide generation in the deep ocean. Annu. Rev. Fluid Mech..

[13] Geveci T (1988). On the application of mixed finite element methods to the wave equation. Math. Model. Numer. Anal.

[14] Hill, D., Griffiths, S., Peltier, W., Horton, B., Törnqvist, T.: High-resolution numerical modeling of tides in the western Atlantic, Gulf of Mexico, and Caribbean Sea during the Holocene. J. Geophys. Res.: Oceans (1978–2012) **116**(C10) (2011)

[15] Holst M, Stern A (2012). Geometric variational crimes: Hilbert complexes, finite element exterior calculus, and problems on hypersurfaces. Found. Comput. Math..

[16] Jayne SR, Laurent LCS (2001). Parameterizing tidal dissipation over rough topography. Geophys. Res. Lett..

[17] Jenkins EW, Rivière B, Wheeler MF (2002). A priori error estimates for mixed finite element approximations of the acoustic wave equation. SIAM J. Numer. Anal..

[18] Kawahara M, Hasegawa K (1978). Periodic Galerkin finite element method of tidal flow. Int. J. Numer. Methods Eng..

[19] Kinnmark IP, Gray WG (1985). Stability and accuracy of spatial approximations for wave equation tidal models. J. Comput. Phys..

[20] Kirby RC, Kieu TT (2015). Symplectic-mixed finite element approximation of linear acoustic wave equations. Numer. Math..

[21] Lamb H (1945). Hydrodynamics.

[22] Le Roux D, Rostand V, Pouliot B (2007). Analysis of numerically induced oscillations in 2D finite-element shallow-water models part I: Inertia-gravity waves. SIAM J. Sci. Comput..

[23] Lefevre F, Lyard F, Provost CL, Schrama EJ (2002). FES99: a global tide finite element solution assimilating tide gauge and altimetric information. J. Atmos. Ocean. Technol..

[24] Leimkuhler B, Reich S (2004). Simulating Hamiltonian Dynamics.

[25] Lynch DR, Gray WG (1979). A wave equation model for finite element tidal computations. Comput. Fluids.

[26] McRae ATT, Cotter CJ (2014). Energy-and enstrophy-conserving schemes for the shallow-water equations, based on mimetic finite elements. Q. J. R. Meteorol. Soc..

[27] Munk W, Wunsch C (1998). Abyssal recipes II: energetics of tidal and wind mixing. Deep-Sea Res. Part I.

[28] Raviart, P.A., Thomas, J.M.: A mixed finite element method for 2nd order elliptic problems. In: Mathematical Aspects of Finite Element Methods (Proc. Conf., Consiglio Naz. delle Ricerche (C.N.R.), Rome, 1975), pp. 292–315. Lecture Notes in Mathematics, vol. 606. Springer, Berlin (1977)

[29] Rognes, M.E., Ham, D.A., Cotter, C.J., McRae, A.T.T.: Automating the solution of PDEs on the sphere and other manifolds in FEniCS 1.2. Geosci Model Dev Discuss **6**(3), 3557–3614 (2013)

[30] Rostand V, Le Roux D (2008). Raviart–Thomas and Brezzi–Douglas–Marini finite-element approximations of the shallow-water equations. Int. J. Numer. Methods Fluids.

[31] Roux DYL (2005). Dispersion relation analysis of the $$P^{NC}_1-P_1$$ finite-element pair in shallow-water models. SIAM J. Sci. Comput..

[32] Roux DYL (2012). Spurious inertial oscillations in shallow-water models. J. Comput. Phys..

[33] Roux DYL, Pouliot B (2009). Analysis of numerically induced oscillations in two-dimensional finite-element shallow-water models part II: Free planetary waves. SIAM J. Sci. Comput..

[34] Salehipour H, Stuhne G, Peltier W (2013). A higher order discontinuous Galerkin, global shallow water model: Global ocean tides and aquaplanet benchmarks. Ocean Model..

[35] Walters RA (2005). Coastal ocean models: two useful finite element methods. Cont. Shelf Res..

[36] Weller H, Ringler T, Piggott M, Wood N (2010). Challenges facing adaptive mesh modeling of the atmosphere and ocean. Bull. Am. Meteorol. Soc..

